# Enhancing Shear Bond Strength of Composite to In-Office Bleached Enamel with Biomimetic Remineralizing Agents: An *In-Vitro* Study

**DOI:** 10.4317/jced.62159

**Published:** 2025-05-01

**Authors:** Sanjana Agarwal, Shashi Rashmi Acharya, Shubha Chhaparwal, Arun Mayya, Akshatha Chatra

**Affiliations:** 1 MDS. Department of Conservative Dentistry and Endodontics, Manipal College of Dental Sciences, Manipal, Manipal Academy of Higher Education, Manipal-576104, Karnataka, India; 2 MDS. Department of Conservative Dentistry and Endodontics, Yenepoya Dental College, Yenepoya (Deemed to be University), 575018, Deralakatte, Mangalore, Karnataka, India

## Abstract

**Background:**

Bleaching procedures alter enamel microstructure, impacting composite bonding. Biomimetic remineralizing agents may restore the bond strength post-bleaching. This in-vitro study aimed to evaluate and compare the role of remineralizing agents on the shear bond strength of composite to in-office bleached enamel using the total-etch technique.

**Material and Methods:**

28 human maxillary premolars were extracted for orthodontic purposes and split into buccal and palatal halves. The 55 samples were divided into five groups. Bleaching procedure was conducted and remineralizing agents were applied twice daily for 5 minutes over 24 days. The five groups included a negative control (Group 1) and groups treated with GC Tooth Mousse Plus (Group 2), Curodont Repair (Group 3), Regenerate Paste (Group 4), and CTx4 Gel (Group 5). A universal composite restorative was applied after total-etch and adhesive application. Shear bond strength was evaluated using an Instron 3366 universal testing machine with a 0.5mm/min loading rate. SEM analysis to assess the failure mode was evaluated on the representative samples. The normality of the data was assessed using the Shapiro-Wilkinson test. Inferential statistics were done using the Whitney U Test (between the groups).

**Results:**

Results of all the test groups (Groups 2-5) had statistically significant differences (*P*< 0.05) when compared with the negative control group (Group 1). There was a significant difference between Group III (Curodont Repair - p11-4 peptide) and Group IV (Regenerate Paste-NR5 Technology) with Group IV being the superior one.

**Conclusions:**

The null hypothesis was rejected. Remineralizing agents enhance the shear bond strength of composite to in-office bleached enamel using the total-etch technique. Regenerate paste (nR5 Technology) showed statistically better results than Curodont Repair paste (p11-4 peptide).

** Key words:**CPP-ACP, Nano-hydroxyapatite, NR-5 Technology, Remineralization, Shear bond strength, Self-assembling peptide P11-4, Tooth bleaching.

## Introduction

The pursuit of aesthetics plays a crucial role in shaping human personality, with patients yearning for a visually pleasing transformation to eradicate any perceived teeth discoloration. In the realm of dental bleaching options, in-office bleaching reigns supreme as the preferred choice due to its ability to deliver noticeable results in a single clinical session, offering a conservative and effective solution. Typically, this procedure entails the application of a potent hydrogen peroxide solution ranging from 20% to 40%, ensuring the attainment of desired outcomes (1).

The impact of bleaching products on enamel characteristics remains a topic of debate within the research community. While some scholars argue that bleaching may induce only minor alterations in tooth structure (2-5), others contend that the effects can be substantial (6-9). The extent of these effects is influenced by factors such as the concentration of hydrogen peroxide in the bleaching agent, the duration of the bleaching procedure, the type and strength of any activators utilized during bleaching, and their presence or absence. Certain studies suggest that bleaching can lead to morphological changes, indicating an erosive process (10). The direct influence of bleaching on the organic protein components of teeth can trigger modifications in the mineral phase, ultimately resulting in observable morphological transformations on the tooth surface.

The aesthetic demands for composites or ceramic veneers over bleached teeth highlight the importance of considering how bleaching can impact the physical and chemical properties of restorative materials, potentially leading to roughness, hardness alterations, cracks, marginal degradation, release of metallic ions, and reduced bond strength to dental structures (8,11). It can also affect the surrounding enamel, compromising interface adhesion (11,12). Concerns arise when restoration is needed after bleaching, as the peroxides in dental substrates can decrease bond strength to enamel by around 60%. This can be attributed to the trapped residual oxygen, which hinders monomer infiltration, polymerization, and thus the bonding mechanism. Therefore, a delay of one to three weeks is recommended before the adhesive restoration procedures take place (13,14).

To address demineralization, novel enamel remineralization technologies have been developed, incorporating biomimetic methods to enhance mechanical properties. While fluoride-containing products aid in remineralization, they may lack the ability to promote ordered apatite crystal growth. Regenerative biomineralization therapy is explored for enamel regeneration, with biomimetic techniques showing promise in restoring enamel microstructure (15,16).

Remineralization materials like Casein phosphopeptide--amorphous calcium phosphate (CPP-ACP), self-assembling peptide (SAP) P11-4, nR5 technology, and nano-hydroxyapatite (n-HAP) offer innovative approaches to enamel repair and regeneration, each with unique mechanisms and benefits.

Casein phosphopeptide--amorphous calcium phosphate (CPP-ACP) and Casein phosphopeptide--amorphous calcium phosphate with fluoride (CPP-ACFP) are nanocomplexes that act as reservoirs of calcium and phosphate, promoting remineralization. CUROLOX® TECHNOLOGY uses SAP P 11-4 peptide, a naturally occurring amino acid, to self-assemble a fibrillar scaffold mimicking the amelogenin enamel matrix. NR-5TM technology enhances saliva mineralization by providing calcium and phosphate, facilitating hydroxyapatite nucleation. Synthetic nanohydroxyapatite (n-HAP) is a biocompatible and bioactive material similar to apatite crystals in enamel, promoting remineralization through synthetic enamel creation or apatite nanoparticle deposition.

Limited data exists on how these remineralizing agents affect composite bond strength to bleached enamel, highlighting the need for further research. Therefore, this in-vitro study aims to evaluate and compare the effect of various remineralizing agents on the shear bond strength of composite resin to in-office bleached enamel using the total-etch technique. By investigating the efficacy of these agents, this study seeks to identify the most effective remineralization strategy for enhancing the bond strength post-bleaching.

## Material and Methods

Following ethical clearance, 28 freshly extracted human maxillary premolars were collected and stored in distilled water. Teeth with intact enamel surfaces were included in the study. The exclusion criteria were teeth with visible or detectable caries, restorations, hyperplastic lesions, stains, cracks, and bleaching.

- Specimen Preparation

The premolars were decoronated using a diamond disk (Horico, Germany). Each crown was sectioned in the mesiodistal plane to obtain two halves, intact buccal and palatal surfaces, giving a total sample size of 55.

A 4mm x 4mm wax sheet was placed on the flattest area of the tooth surface, and the surrounding area was coated with acid-resistant nail varnish. After drying, the wax sheet was removed, and the specimens were ready for treatment. Throughout the preparation, specimens were stored in 0.1% thymol anti-fungal solution. Pulpal tissues were removed using a barbed broach, and the pulp chamber was sealed with pink wax.

- Bleaching Procedure

Chemically activated Ultradent Opalescence Boost Pf 40% gel was applied from a dual barrel syringe in a 1.5-2mm thick layer. The gel was left on the tooth surface undisturbed for 15 minutes before being removed with a dry cotton swab. This application process was repeated three times, totaling a 45-minute application time.

- Evaluated Groups

The 55 samples were randomly divided into 5 groups, with each group consisting of 11 samples:

Group 1:(n=11) Negative control with no remineralizing agent used post-bleaching and restored with composite using total-etch technique.

Group 2: (n=11) GC Tooth Mousse Plus (CPP-ACP+Fluoride) 

Group 3: (n=11) Curodont Repair ( P 11-4 peptide) 

Group 4: (n=11) Regenerate (nR5 technology) 

Group 5: (n=11) CTx4 Gel (n-HAP + Fluoride + Xylitol) 

These remineralizing agents were applied post-bleaching and restored with composite using the total-etch technique.

- Application of Remineralizing Pastes

Specimens were dried with a cotton pellet before the application of each remineralizing agent, and a thin layer of the assigned remineralizing agent was applied using a cotton tip applicator.

Each remineralizing agent was applied to the enamel of the assigned specimens for 5 minutes twice a day. These procedures were repeated for 24 sequential days with a total time of four hours. The enamel did not receive treatment with the remineralizing agents for the negative control group. Between the application of remineralizing agents, the sample area was washed with deionized water and kept in artificial saliva prepared at room temperature (27°C)

Custom-made metal moulds were prepared, and acrylic cold-cure resin (DPI-RR Cold Cure, DPI, Mumbai, India) was poured vertically by positioning the sample.

- Restorative Procedure

Etchant containing 37% Phosphoric Acid was applied to the sample area for 15 seconds and then washed. The bonding agent (Adper Single Bond 2) was then applied with an applicator tip and cured.

After application of the adhesive, a standard polyvinyl chloride tube with an internal diameter of 4 mm and a height of 4 mm was placed perpendicularly on the enamel surface and the resin composite (FiltekTM Z250 Universal Restorative (3M ESPE, Seefeld, Germany) was carefully inserted into the tube and cured. The specimens were stored in artificial saliva for 48 hours at room temperature (27°C) before shear bond strength testing.

- Bond Strength

All samples were evaluated using the universal testing machine (Instron 3366) with a loading rate at 0.5mm/min for shear bond strength evaluation. All samples were evaluated under stereomicroscope while representative samples were used for SEM analysis to check for Adhesive/Cohesive/Mixed Failure.

- Statistical Analysis

Data was analyzed using the statistical package SPSS 23.0 (SPSS Inc., Chicago, IL), and the level of significance was set at *P*<0.05. Descriptive statistics was performed to assess the mean and standard deviation of the respective groups. The normality of the data was assessed using the Shapiro-Wilkinson test. Since the data did not follow normality, inferential statistics were used to determine the difference between the groups using the Mann-Whitney U Test.

## Results

**Figure 1:**
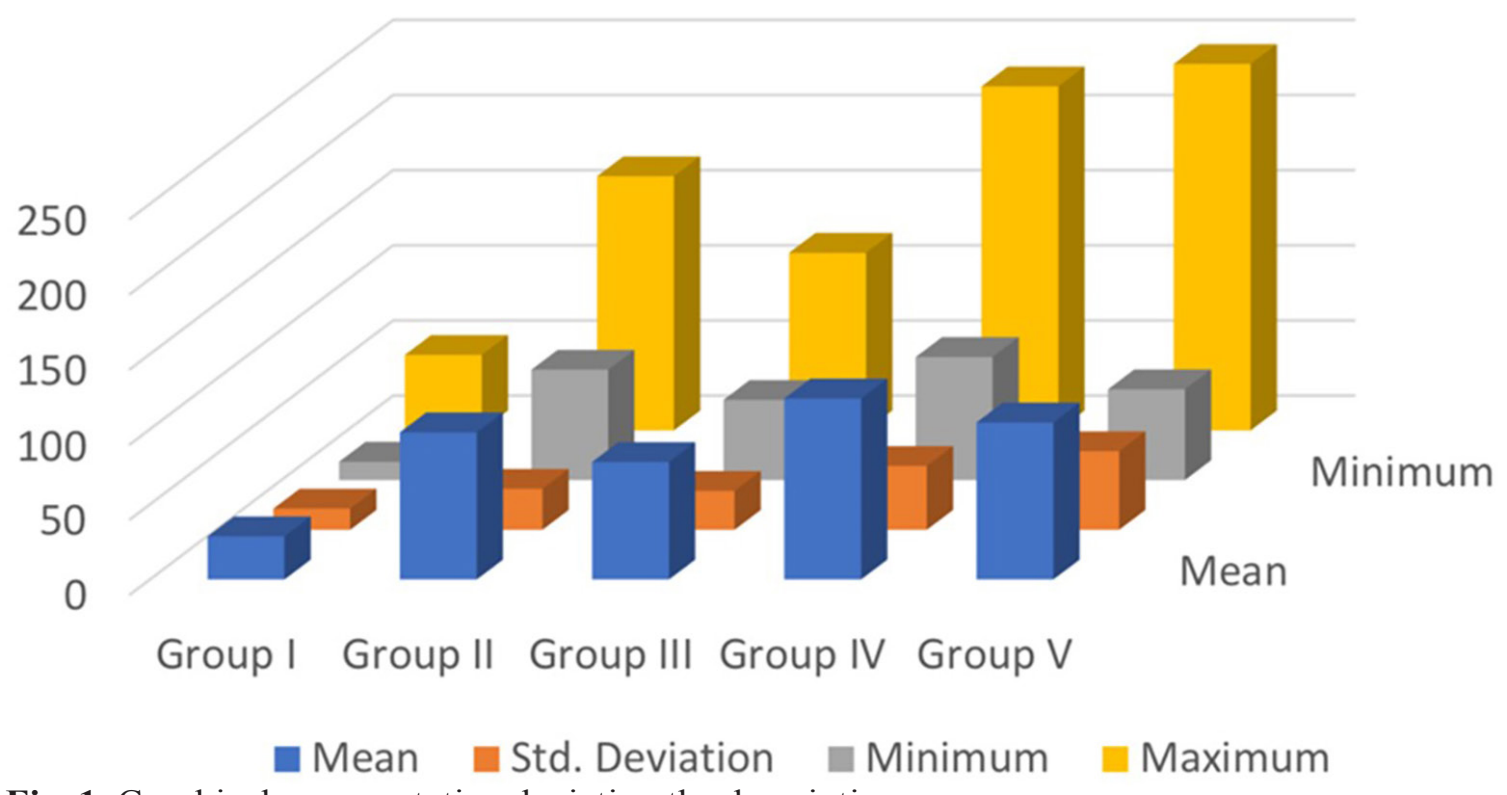
Graphical representation depicting the descriptives.

**Figure 2:**
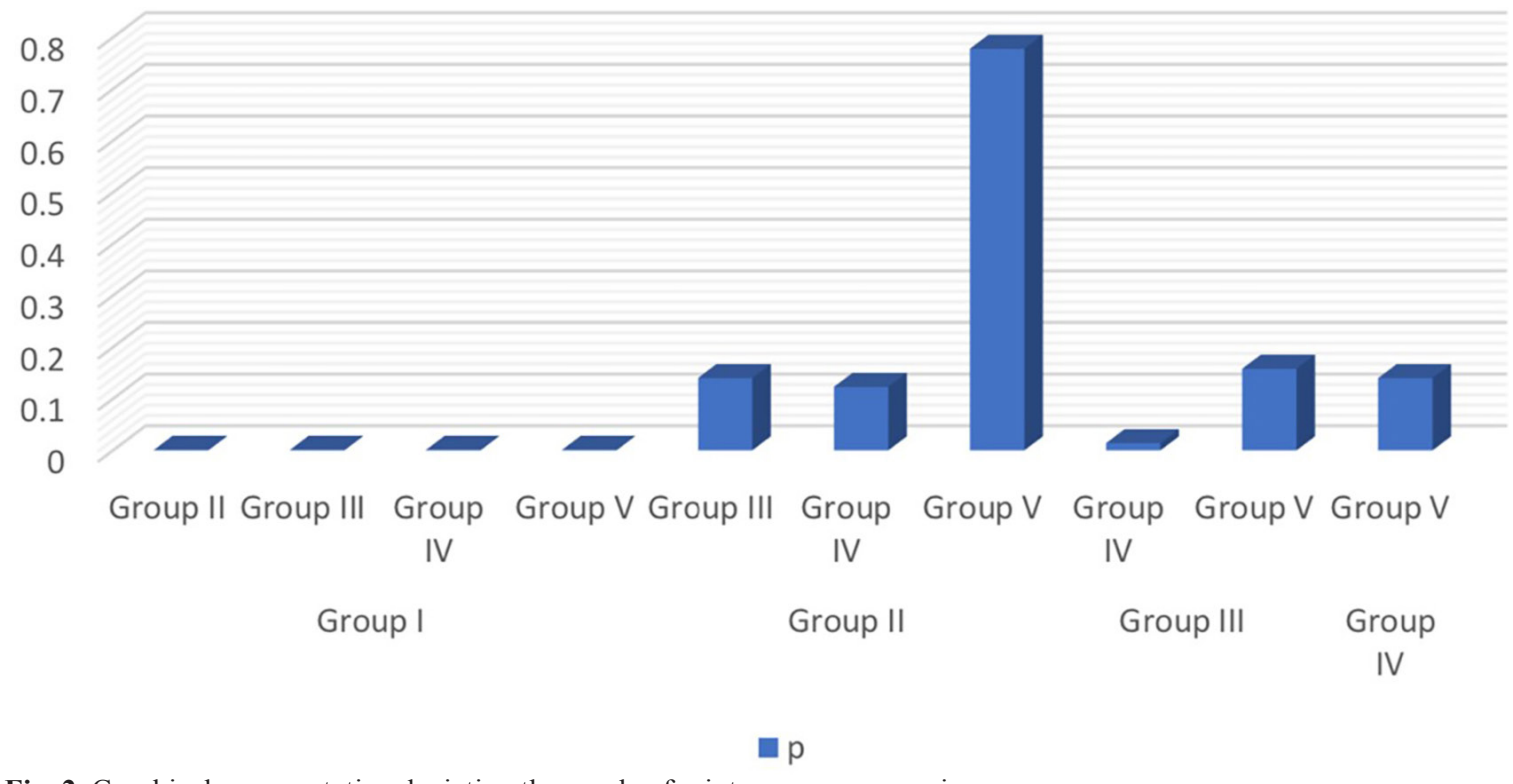
Graphical representation depicting the *p*-value for inter-group comparison.

 Table 1 and Figure 1 depict the description of each group.  Table 2 depicts a statistically significant difference (*P* < 0.05) between the control group (Group I) and test groups (Group II-V). Also, there is a statistical difference between Group III and Group IV. The representation of the above data is depicted in Figure 2.

 Table 3 depicts the distribution of the type of failure noticed in the specimens of each group. The Chi-square test proved no statistical difference between the groups, concerning the mode of failure

**Figure 3:**
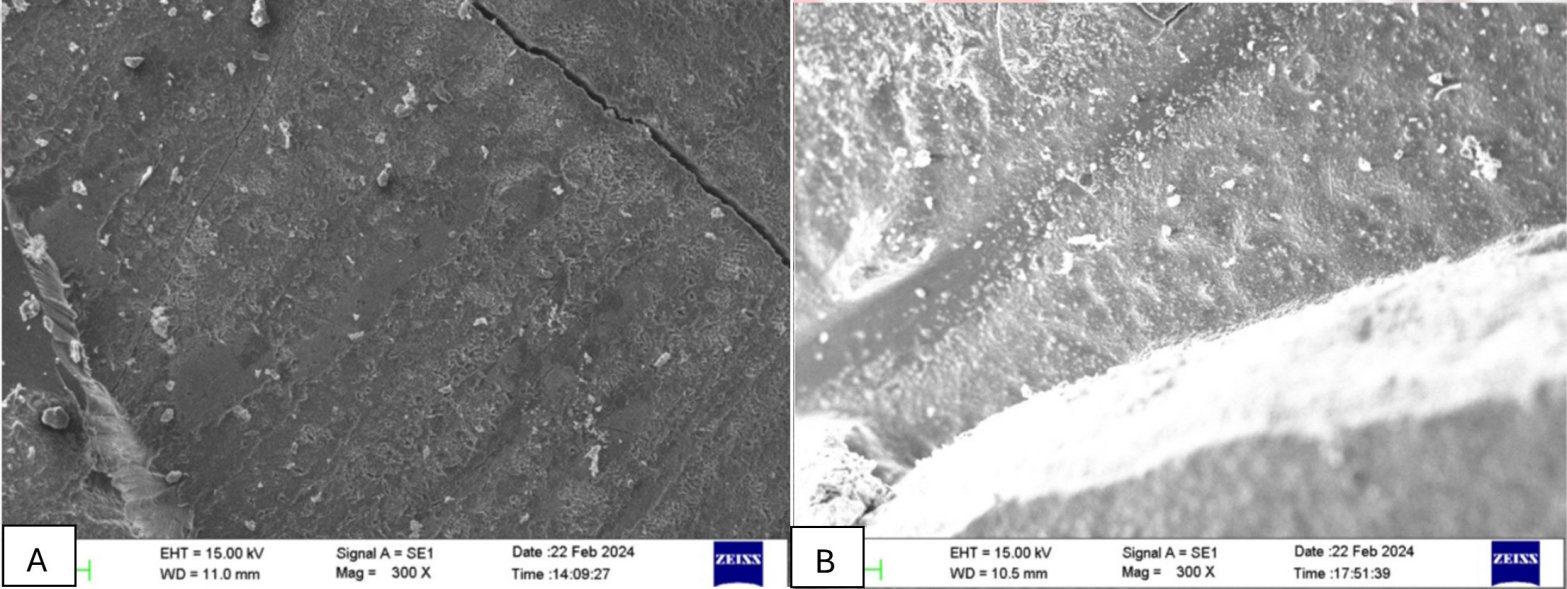
Scanning Electronic Microscopy (SEM) images of representative samples depicting different modes of failure: (A) Adhesive Failure; (B) Cohesive Failure.

In the SEM analysis (Fig. 3), the control group (Fig. 3A) showed significant enamel surface degradation, including visible cracks and porosity. In contrast, the group treated with Regenerate paste (Fig. 3B) exhibited a smoother surface with reduced porosity and improved structural integrity. These findings align with the mechanical test results, where the Regenerate paste significantly enhanced the shear bond strength by providing better protection and remineralization of the bleached enamel surface.

## Discussion

Biomimetic materials can potentially rebuild damaged bleached enamel (17), leading to the rejection of the null hypothesis in the study. The dynamic bleaching process involves peroxide penetration, interaction with chromogen, and alteration of enamel properties, influenced by product concentration and exposure duration. Despite concerns raised by the literature, bleaching agents containing a high concentration of hydrogen peroxide are being prescribed, applied, and reapplied multiple times during the same clinical session to expedite tooth color change (18).

Bleached surfaces may require composite/ceramic veneers for improved appearance. The bond between tooth surface and restorative material may be reduced due to delayed oxygen release, preventing resin penetration or polymerization. The bond strength reverts to normal if the oxygen-rich enamel layer is eliminated, which must be greater than 5-10 μm, as the acid etching procedure would have eliminated it otherwise (19).

The current investigation compared the conventional posology based on three applications of a 35-40% HP-based solution. The most optimal bleaching period, as established by Ozdemir Z and Surmelioglu D in their investigation, was found to be 40 minutes, which was almost similar to the current study, where we used 45 minutes (20).

Bleaching substances can break down enamel and dentin matrix, leading to oxidation-reduction reactions. Remineralizing agents, combined with saliva, may help restore the mineral component of enamel (21). This study used artificial saliva to replicate clinical settings. Biomimetic toothpastes can enhance oral health by encouraging remineralization and fortifying teeth. Post-bleaching therapies that can remineralize the enamel surface are highly recommended (17).

The present study explores the use of biomimetic remineralizing pastes for guided enamel regeneration, a therapeutic approach that can reduce the long-term damaging effects of tooth bleaching. Given that each of the biomimetic remineralizing pastes selected for this investigation has a distinct mode of action, while there is insufficient data available for comparison in the current literature, the present research is unique. The present research confirms that remineralizing agents improve the shear bond strength of composites to bleached enamel, contradicting the null hypothesis. Comparable results were concluded in myriad studies.

CPP-ACP (Casein Phosphopeptides-Amorphous Calcium Phosphate) is a complex formed by phosphorylated casein-derived peptides that bind to ACP nanoclusters, preventing their growth to a critical size necessary for nucleation and precipitation. These complexes diffuse into subsurface enamel lesions, creating a supersaturated environment of calcium and phosphate ions, enhancing fluoride effectiveness in remineralization (22).

CUROLOX® TECHNOLOGY utilizes self-assembling peptide P11-4, designed to mimic the amelogenin enamel matrix. This peptide forms a biocompatible scaffold that triggers hydroxyapatite precipitation from saliva ions, increasing the Ca: *P* ratio and inhibiting mineral loss (23,24).

NR-5™ technology combines calcium silicate, sodium phosphate salts, and fluoride to enhance natural mineralization by releasing calcium from Calcium Silicate (CaSi), facilitating hydroxyapatite nucleation for enamel remineralization (25,26).

Nano hydroxyapatite (n-HAP) is a biocompatible material that resembles tooth enamel crystals. During remineralization, n-HAP crystals fill defects and micropores in demineralized enamel, offering a biomimetic repair option (27,28).

The study found that Regenerate paste had the highest mean shear bond strengths (120.445 MPa), followed by n-HAP (104.513 MPa), CPP-ACP (97.952 MPa), and Curodont Repair P 11-4 peptide (78.195 Mpa). The negative control group had the least bond strength (28.686 Mpa). Regenerate paste (Group IV) significantly increased microhardness, while Curodont Repair paste (Group II) had the lowest bond strength values.

The superior effects of the Regenerate paste can be attributed to its mode of action, which includes multiple mechanisms. There is a dearth of evidence regarding the interaction of the P11-4 peptide on the bleached surface and its implications on the bond strength values. The inferior results of Curodont paste may be due to the time-dependent process of remineralization with the peptide, which may require multiple administrations over 3-6 months (29). Attin *et al*. observed no anti-erosive properties of the P11-4 peptide (30), while Brunton *et al*. contradictorily concluded that even a single application of Curodont repair leads to remineralization (23).

The study found significant variation in shear bond strength across different enamel groups, possibly due to the composition of remineralizing agents and resin-based composite materials used. The most common failure mechanism was adhesive enamel failure (45.5%), followed by mixed enamel failure (40%) and cohesive enamel failure (14.5%). No significant variation was found in the correlation between these failure categories and the various groups (31).

Scanning Electron Microscopy (SEM) microphotographs (Fig. 3) revealed that the formation of a protective layer on bleached enamel was unique among these agents. SEM of specimens treated with remineralizing agents showed different findings, including smooth intact surfaces for unmineralized enamel, interprismatic dissolution of enamel, porosity, prism irregularity, exposed underlying perikymata, and deepened tomes process in demineralized enamel, similar to previous findings (32).

This study was conducted in an *in vitro* setting, straight extrapolations to clinical situations must be done with caution. The samples were kept in artificial saliva solution for 4 hours, which may not provide long-term effects. Since a prior study showed that thermocycling had no effect on the shear bond strength of the tested materials to enamel and dentin (31), thermocycling of the specimens was not done in this investigation.

The study showed a strong link between the effects of remineralization materials and shear bond strength. The experimental groups had biomimetic properties while also having distinct processes; nonetheless, there is a dearth of comparative data in the literature, making the current study noteworthy. Despite the limitations, the present research highlights several beneficial connections between *in vitro* effectiveness and the short-term impact of remineralizing agents on the clinical efficacy of shear bond strength of composites on bleached enamel.

## Conclusions

With the limitations of the study, the null hypothesis was rejected drawing the following conclusions:

1. Application of remineralizing pastes significantly improved the shear bond strength of composite to in-office bleached enamel using the total-etch technique.

2. The application of Regenerate paste improved the shear bond strength of composite to in-office bleached enamel using the total-etch technique better than the Curodont paste.

## Figures and Tables

**Table 1:** Descriptives showing the Maximum and Minimum force in Newton (N), the Mean and Standard Deviation of each group with a sample size(n) of 11.

Groups	n	Mean^a^	Std.Deviation	Minimum [N]	Maximum [N]
Group I	11	28.686	14.090	12.200	50.200
Group II	11	97.952	27.293	73.460	169.100
Group III	11	78.195	25.921	53.370	118.170
Group IV	11	120.445	42.492	81.920	228.870
Group V	11	104.513	52.279	60.430	243.830

a. H=30.773 *p*<0.001; H- Kruskal Wallis test

**Table 2:** Inter-Group Comparison Results.

Group	Group	Mean Difference	Mann-Whitney U test (Z)	p*
Group I	Group II	-69.265	3.973	<0.001 VHS
	Group III	-49.509	3.973	<0.001 VHS
	Group IV	-91.758	3.973	<0.001 VHS
	Group V	-75.826	3.973	<0.001 VHS
Group II	Group III	19.756	1.477	0.14 NS
	Group IV	-22.492	1.543	0.123 NS
	Group V	-6.560	0.381	0.778 NS
Group III	Group IV	-42.249	2.482	0.014 SIG
	Group V	-26.317	1.412	0.158 NS
Group IV	Group V	15.931	1.477	0.14 NS

*p<0.05 is statistically significant (Mann-Whitney U test) VHS-Very highly significant; SIG-Significant; NS-Not Significant.

**Table 3:** Distribution of the type of failure noticed in the specimens of each group.

		GROUP I	GROUP II	GROUP III	GROUP IV	GROUP V	TOTAL
ADHESIVE	Count	8	3	5	4	5	25
	%	72.7%	27.3%	45.5%	36.4%	45.5%	45.5%
MIXED	Count	1	6	4	6	5	22
	%	9.1%	54.5%	36.4%	54.5%	45.5%	40.0%
COHESIVE	Count	2	2	2	1	1	8
	%	TOTAL	18.2%	18.2%	9.1%	9.1%	14.5%
TOTAL	Count	11	11	11	11	11	55
	%	100.0%	100.0%	100.0%	100.0%	100.0%	100.0%

The Chi-square test proved no statistical difference between the groups concerning the mode of failure. Chi-Square test (X2)=7.459 *p*=0.488 (not significant)

## Data Availability

The datasets used and/or analyzed during the current study are available from the corresponding author.
